# Association between platelet indices and VCAM-1 levels in patients with coronary artery disease

**DOI:** 10.1016/j.ahjo.2026.100960

**Published:** 2026-10-14

**Authors:** Ariosta, Banundari Rachmawati, Udin Bahrudin, N.S. Widyastiti, Dwi Retnoningrum

**Affiliations:** a Doctoral study Program of Medical and Health Science, Faculty of Medicine, Diponegoro University, Indonesia; b Department of Clinical Pathology, Faculty of Medicine, Diponegoro University, Indonesia; c Department of Cardiology and Vascular Medicine, Faculty of Medicine, Diponegoro University, Indonesia

**Keywords:** VCAM-1, platelet count, mean platelet volume, P-LCR, large platelets, endothelial activation, coronary artery disease

## Abstract

**Background:**

Routine platelet indices reflect platelet size and turnover, whereas soluble vascular cell adhesion molecule-1 (sVCAM-1) reflects endothelial activation. Whether these inexpensive indices relate to circulating VCAM-1 in coronary artery disease (CAD) is unclear.

**Objective:**

To evaluate the association between platelet indices and sVCAM-1 in patients with CAD.

**Methods:**

This cross-sectional study consecutively enrolled 80 patients with stable, angiographically confirmed CAD (>50% stenosis in at least one major coronary artery) at Dr. Kariadi General Hospital, Semarang, Indonesia. Platelet count, mean platelet volume (MPV), platelet large cell ratio (P-LCR) and large-platelet count were measured with a Sysmex XN-1000 analyser, and serum sVCAM-1 by sandwich ELISA (Elabscience). Spearman correlation and multivariable linear regression were used.

**Results:**

Platelet count (ρ = 0.24, *p* = 0.036) and large-platelet count (ρ = 0.35, *p* = 0.002) were weakly correlated with sVCAM-1 (344.23 ± 144.98 ng/mL), whereas MPV (ρ = 0.10, *p* = 0.387) and P-LCR (ρ = 0.11, *p* = 0.336) were not. After adjustment for clinical covariates, only large-platelet count remained associated with sVCAM-1 (β = 58.7 ng/mL per SD; 95% CI 23.4–94.1; *p* = 0.002).

**Conclusion:**

In stable CAD, large-platelet count and, less robustly, platelet count, but not MPV or P-LCR, were weakly associated with sVCAM-1. These hypothesis-generating findings require confirmation in larger prospective studies.

## 1 Introduction

Coronary artery disease (CAD) is still one of the leading causes of cardiovascular morbidity and mortality globally. It is a progressive condition characterised by the buildup of plaque within the coronary arterial walls. The development and progression of atherosclerosis are closely linked to endothelial dysfunction and platelet activation, which play pivotal roles in this process. Platelet indices and VCAM-1 have both been proposed as markers related to these processes [1], [2].

Platelet indices are indirect markers of platelet size, production and reactivity. Larger platelets, which are generally younger, contain more granules and are more adhesive and prone to aggregation. Large platelets are also enzymatically and metabolically more active compared to smaller ones [2]. Large platelets are therefore considered an indirect indicator of platelet reactivity [3]. Additionally, the size of platelets can be measured using the mean platelet volume (MPV) [4], [5]. MPV represents the average size of platelets in circulation and can be utilized as an index to evaluate platelet size and activity [6]. Larger platelets contain more granules and produce higher amounts of thromboxane A2, which enhances their aggregation response to collagen and expression of glycoprotein Ib and IIb/IIIa receptors [7], [8]. This results in higher chance of thrombus formation in individuals with elevated MPV [9].

VCAM-1 is a marker of endothelial activation. It belongs to the immunoglobulin superfamily of adhesion molecules, has a molecular weight of 100–110 kDa, and mediates leukocyte–endothelial adhesion and signalling, which is crucial in the development of atherosclerosis [10]. Increased endothelial production of cellular adhesion molecules (CAMs) contributes to arterial plaque development [11], [12], [13], [14].

Platelets and the endothelium interact continuously during atherogenesis. Upon activation, platelets translocate P-selectin to their surface, release CD40 ligand (CD40L), interleukin-1β and chemokines, and shed platelet-derived microparticles; these mediators can activate endothelial cells and up-regulate adhesion molecules, including VCAM-1 [15], [16], [17], [18]. Conversely, an inflamed, dysfunctional endothelium promotes platelet activation and consumption, which may stimulate the production of younger, larger platelets [9], [19]. VCAM-1 is of particular interest in this context because it is cytokine-inducible, mediates firm adhesion and transmigration of monocytes and lymphocytes through very late antigen-4 (VLA-4), and is expressed early in atherosclerotic lesion formation [12], [20]. However, it remains unclear whether the routine platelet indices reported by automated haematology analysers, which are inexpensive and universally available, reflect this platelet–endothelial axis in patients with established CAD. Previous studies have evaluated platelet indices mainly in relation to the presence, severity or outcome of CAD [4], [19], [21], and soluble adhesion molecules mainly as prognostic markers [14], [22]; to our knowledge, few studies have examined the two together.

The aim of this study was to evaluate the correlation between platelet indices—platelet count, mean platelet volume (MPV), platelet large cell ratio (P-LCR) and absolute large-platelet count—and circulating soluble VCAM-1 (sVCAM-1) levels in patients with angiographically confirmed CAD. We hypothesised that indices reflecting a larger or more abundant platelet mass would be positively correlated with sVCAM-1 levels.

## 2 Methodology

### 2.1 Study design and participants

This observational analytic study used a cross-sectional design and was conducted at Dr. Kariadi General Hospital, Semarang, Indonesia, from March to July 2021. Patients with stable CAD attending the hospital during the study period were enrolled consecutively. CAD was defined as >50% luminal stenosis in at least one of the three major coronary arteries (left anterior descending, left circumflex or right coronary artery) on coronary angiography; patients without such a lesion were not enrolled. All patients had stable CAD; patients with acute coronary syndrome were not included. The number of diseased vessels (one-, two- or three-vessel disease) was recorded. Participants were drawn from the same parent cohort as a previous report on sVCAM-1 and absolute monocyte count, which analysed 74 of these patients after exclusion of six patients with extreme values [23]. The study was approved by the Health Research Ethics Committee of Dr. Kariadi General Hospital (No. 791/EC/KEPK-RSDK/2021), and written informed consent was obtained from all participants.

### 2.2 Eligibility criteria

Patients aged 30–65 years with angiographically confirmed CAD were eligible. Exclusion criteria were haematological malignancy, thrombocytopenia (platelet count <100 × 10^3^/μL), leukocytosis (leukocyte count >11 × 10^3^/μL). Demographic data, smoking, diabetes mellitus, body weight and height, blood pressure, HbA1c, serum creatinine and medication use at the time of sampling (aspirin, P2Y12 inhibitors, statins, ACE inhibitors, angiotensin receptor blockers, β-blockers and nitrates) were obtained from interviews, physical examination and medical records.

### 2.3 Blood sampling and platelet indices

Venous blood was collected on the day of coronary angiography or the following day. Platelet indices were measured in K3-EDTA whole blood within 2 h of collection on a Sysmex XN-1000 automated haematology analyser (Sysmex Corporation, Kobe, Japan) at the Clinical Pathology Laboratory of Dr. Kariadi General Hospital, using the impedance (sheath-flow direct-current) method. MPV is the mean volume of the whole platelet population, whereas P-LCR is the analyser-defined percentage of platelets with a volume greater than 12 fL; the 12 fL threshold is a fixed, manufacturer-defined parameter of this analyser rather than a study-specific cut-off. The absolute large-platelet count was calculated as P-LCR × platelet count / 100. Internal quality control was performed daily according to the laboratory's standard procedures.

### 2.4 Measurement of soluble VCAM-1

The analyte measured was soluble (circulating) VCAM-1, not endothelial surface-bound VCAM-1. Blood was collected in plain tubes and centrifuged to obtain serum; microparticle-free plasma was not prepared. Serum was stored at −20 °C for a maximum of 5 months until analysis; samples were not refrozen and were thawed only once, immediately before all samples were assayed in a single batch. sVCAM-1 was measured at the Iodine Deficiency Disorders (GAKY) Laboratory, Faculty of Medicine, Universitas Diponegoro, using a commercial sandwich ELISA kit (Human VCAM-1/CD106 ELISA Kit, cat. no. E-EL-H5587; Elabscience Biotechnology, Wuhan, China; detection range 1.56–100 ng/mL; analytical sensitivity 0.94 ng/mL; manufacturer-reported intra- and inter-assay coefficients of variation 4.38–5.60% and 4.18–5.51%, respectively) according to the manufacturer's instructions, and absorbance was read on a BioTek ELx800 microplate reader (BioTek Instruments, Winooski, VT, USA). Samples were diluted 1:10 and measured in singlicate.

### 2.5 Statistical analysis

Analyses were performed using Python 3.12 (Python Software Foundation) with the pandas 3.0, SciPy 1.17 and statsmodels 0.15 libraries. Continuous variables are presented as mean ± standard deviation and median (minimum–maximum), and categorical variables as number (percentage). Body mass index was calculated as weight/height [2], and estimated glomerular filtration rate (eGFR) with the 2021 CKD-EPI creatinine equation. Normality was assessed using the Shapiro–Wilk test, complemented by histograms and Q–Q plots. Because sVCAM-1 and all platelet indices deviated from normality (all Shapiro–Wilk *p* < 0.05) and transformation did not normalise them, correlations were assessed with Spearman's rank correlation (ρ) on untransformed data. Correlation strength was interpreted as negligible (|ρ| < 0.10), weak (0.10–0.39), moderate (0.40–0.69) or strong (≥0.70) [24]. To adjust for confounding, multivariable linear regression was performed with sVCAM-1 as the dependent variable and each platelet index entered separately (standardised per 1 SD), adjusted for age, sex, BMI, smoking, diabetes mellitus, eGFR, three-vessel disease and P2Y12 inhibitor use; a second model additionally included leukocyte count as a marker of systemic inflammation. Statin use was not entered because all patients received atorvastatin. Model residuals were approximately normally distributed (Shapiro–Wilk *p* = 0.45). In a sensitivity analysis, analyses were repeated after excluding five patients with sVCAM-1 < 100 ng/mL, values far below the rest of the distribution (next lowest value 124 ng/mL). A two-sided *p* < 0.05 was considered statistically significant.

## 3 Results

A total of 80 patients with coronary artery disease (CAD), diagnosed through angiography, were included. Of the 80 patients, 63 (78.8%) were male and 17 (21.2%) were female, with a mean age of 56.96 ± 5.38 years (range 42–65). Nearly half had three-vessel disease (48.8%), one-third had diabetes mellitus (33.8%), and all received atorvastatin; 91.2% received aspirin and 72.5% a P2Y12 inhibitor (Table 1).

**Table 1:** Distribution of study data characteristics.

Variable	Mean ± SD or n (%)	Median (min-max)
Sex - Male = 63 (78.8%) - Female = 17 (21.2%)		
Age (years)	56.96 ± 5.38	58 (42–65)
BMI (kg/m^2^)	26.01 ± 3.47	25.48 (19.83–35.08)
Smoking, n (%)	39 (48.8%)	–
Diabetes mellitus, n (%)	27 (33.8%)	–
HbA1c (%), *n* = 66	6.75 ± 1.77	6.35 (4.0–15.1)
Systolic blood pressure (mmHg)	126.85 ± 20.82	121.5 (80–170)
Diastolic blood pressure (mmHg)	76.62 ± 12.42	79.5 (42–102)
Serum creatinine (mg/dL)	1.26 ± 0.34	1.20 (0.70–2.60)
eGFR (mL/min/1.73 m^2^)	67.70 ± 18.64	67.23 (24.79–108.49)
Haemoglobin (g/dL)	13.68 ± 1.77	13.80 (9.50–18.60)
Leukocyte count (10^3^/μL)	7.92 ± 1.64	7.80 (4.34–12.02)
Diseased vessels, n (%)		
One-vessel disease	20 (25.0%)	–
Two-vessel disease	21 (26.2%)	–
Three-vessel disease	39 (48.8%)	–
Aspirin, n (%)	73 (91.2%)	–
Clopidogrel, n (%)	35 (43.8%)	–
Ticagrelor, n (%)	25 (31.2%)	–
Dual antiplatelet therapy, n (%)	55 (68.8%)	–
Atorvastatin, n (%)	80 (100%)	–
ACE inhibitor, n (%)	43 (53.8%)	–
Angiotensin receptor blocker, n (%)	27 (33.8%)	–
β-Blocker, n (%)	68 (85.0%)	–
Nitrate, n (%)	66 (82.5%)	–
Platelets (10^3^/μL)	282.19 ± 84.91	280.5 (128–592)
MPV (fL)	10.31 ± 0.95	10.15 (8.7–13)
P-LCR (%)	27.20 ± 7.84	25.70 (15.50–48.40)
Large platelets (10^3^/uL)	73.82 ± 21.85	71.24 (37.76–128.62)
sVCAM-1 (ng/mL)	344.23 ± 144.98	358.50 (32–604)

The mean platelet count was 282.19 ± 84.91 × 10^3^/μL (range 128–592 × 10^3^/μL). The mean platelet volume (MPV) was 10.31 ± 0.95 fL (range 8.7–13.0 fL).

P-LCR represents the percentage of platelets larger than 12 fL. The mean P-LCR was 27.20 ± 7.84% (range 15.50–48.40%). The absolute large-platelet count (platelets >12 fL), calculated as P-LCR × platelet count, was 73.82 ± 21.85 × 10^3^/μL (range 37.76–128.62 × 10^3^/μL).

VCAM-1 levels were measured using the ELISA method at the GAKY Laboratory. The mean VCAM-1 level in CAD patients was 344.23 ± 144.98 ng/mL, with a range of 32 to 604 ng/mL (Table 1).

Normality was assessed with the Shapiro–Wilk test. Platelet count (*p* = 0.004), MPV (*p* = 0.001), P-LCR (*p* = 0.002), large-platelet count (*p* = 0.024) and sVCAM-1 (*p* = 0.021) all deviated from a normal distribution, and transformation did not achieve normality; therefore, Spearman's rank correlation was used for all correlation analyses.

Platelet count showed a statistically significant but weak positive correlation with sVCAM-1 (ρ = 0.235, *p* = 0.036; Table 2, Fig. 1A).

**Table 2:** Statistical test for the relationship between platelet indices and VCAM-1 levels.

Variable	VCAM-1	
	p	ρ
Platelets	0.036	0.235
MPV	0.387	0.098
P-LCR	0.336	0.109
Large platelets	0.002	0.346

Note: all analyses used Spearman's rank correlation on untransformed data (*n* = 80). ρ, Spearman correlation coefficient.

**Fig. 1:**
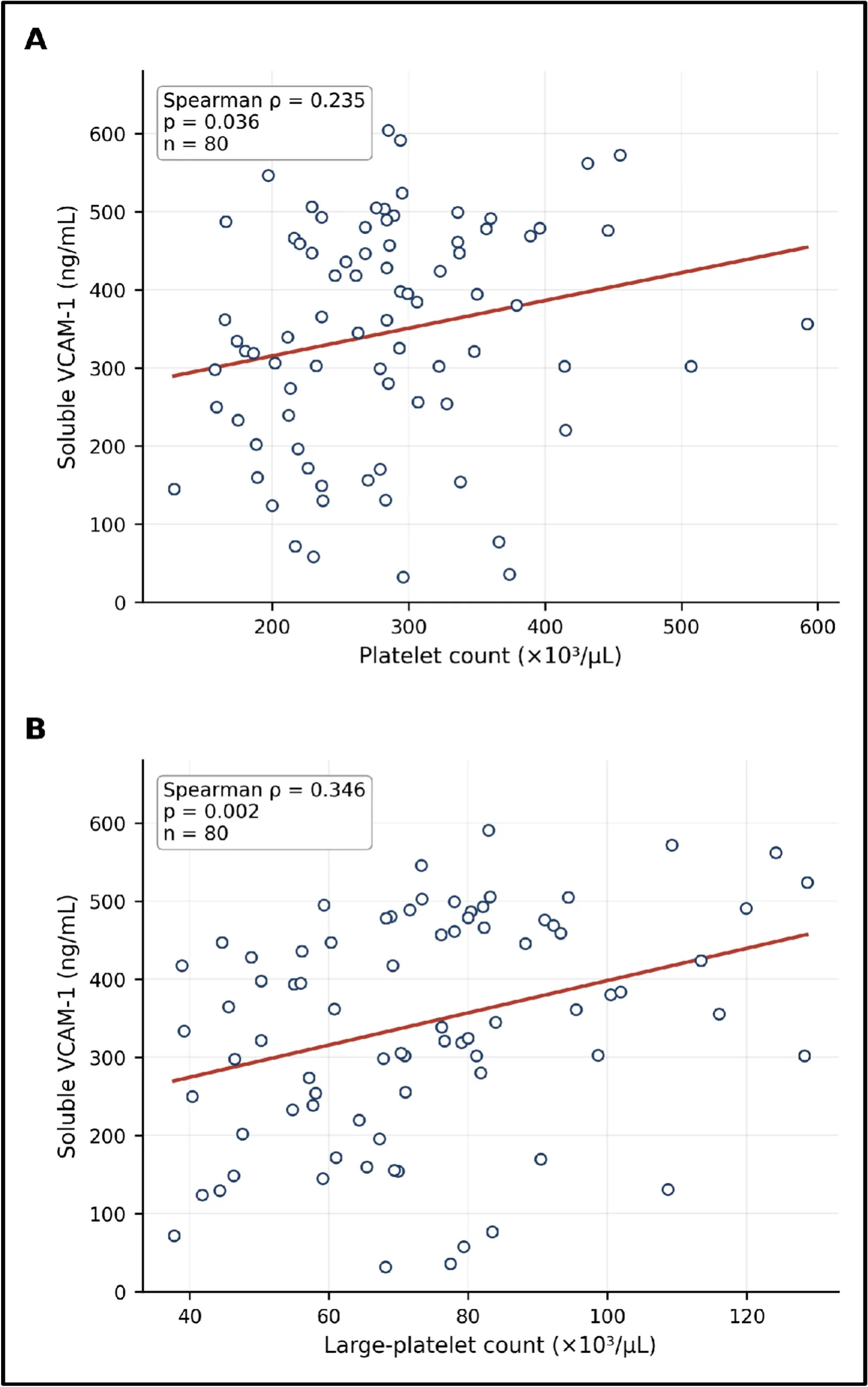
Scatter plots of (A) platelet count and (B) absolute large-platelet count against soluble VCAM-1 in 80 patients with stable CAD. Each point represents one patient; the line is the least-squares regression line, shown for visual guidance. Spearman's rank correlation: (A) ρ = 0.235, *p* = 0.036; (B) ρ = 0.346, *p* = 0.002.

The MPV and PLCR values did not show a significant relationship with VCAM-1 levels. Both showed negligible to weak, non-significant correlations (MPV: ρ = 0.098, *p* = 0.387; P-LCR: ρ = 0.109, *p* = 0.336; Table 2).

Large-platelet count showed a weak positive correlation with sVCAM-1 (ρ = 0.346, *p* = 0.002; Table 2, Fig. 1B). Although large-platelet count is derived from platelet count, its association with sVCAM-1 persisted after controlling for platelet count (partial ρ = 0.27, *p* = 0.015), whereas that of platelet count did not persist after controlling for large-platelet count (partial ρ = 0.07, *p* = 0.54).

In multivariable analysis (Table 3), large-platelet count remained independently associated with sVCAM-1 after adjustment for age, sex, BMI, smoking, diabetes, eGFR, three-vessel disease and P2Y12 inhibitor use (β = 58.7 ng/mL per SD; 95% CI 23.4–94.1; *p* = 0.002) and after further adjustment for leukocyte count (β = 44.7; 95% CI 5.9–83.5; *p* = 0.024). The association of platelet count was attenuated after adjustment (β = 30.2; 95% CI −3.4 to 63.9; *p* = 0.078) and disappeared after adjustment for leukocyte count (*p* = 0.421); platelet count was itself correlated with leukocyte count (ρ = 0.36, *p* = 0.001), which was also correlated with sVCAM-1 (ρ = 0.35, *p* = 0.001). MPV and P-LCR were not associated with sVCAM-1 in any model. Among the covariates, patients with diabetes had higher sVCAM-1 than those without (393.7 ± 144.8 vs 319.0 ± 139.7 ng/mL; Mann–Whitney *p* = 0.016).

**Table 3:** Multivariable linear regression of sVCAM-1 on platelet indices (*n* = 80).

Platelet index (per 1 SD)	Unadjusted β (95% CI)	p	Model 1^a^ β (95% CI)	p	Model 2^b^ β (95% CI)	p
Platelet count (SD 84.9 × 10^3^/μL)	30.1 (−1.9 to 62.1)	0.065	30.2 (−3.4 to 63.9)	0.078	14.4 (−21.0 to 49.7)	0.421
MPV (SD 0.95 fL)	11.2 (−21.4 to 43.8)	0.495	15.7 (−18.7 to 50.1)	0.365	16.0 (−16.8 to 48.8)	0.334
P-LCR (SD 7.8%)	13.7 (−18.8 to 46.2)	0.405	18.4 (−16.0 to 52.9)	0.290	18.9 (−13.9 to 51.7)	0.253
Large-platelet count (SD 21.9 × 10^3^/μL)	45.1 (14.0 to 76.1)	0.005	58.7 (23.4 to 94.1)	0.002	44.7 (5.9 to 83.5)	0.024

β, change in sVCAM-1 (ng/mL) per 1-SD increase in the platelet index; each index was modelled separately (*n* = 80).

a Model 1: adjusted for age, sex, BMI, smoking, diabetes mellitus, eGFR, three-vessel disease and P2Y12 inhibitor use.

b Model 2: Model 1 plus leukocyte count. CI, confidence interval.

In the sensitivity analysis excluding five patients with sVCAM-1 < 100 ng/mL (*n* = 75), the correlations were slightly stronger (platelet count ρ = 0.307, *p* = 0.007; large-platelet count ρ = 0.380, *p* < 0.001; MPV ρ = 0.050, *p* = 0.672; P-LCR ρ = 0.066, *p* = 0.573), and both platelet count (*p* = 0.024) and large-platelet count (*p* = 0.003) were associated with sVCAM-1 in the adjusted model.

## 4 Discussion

Coronary artery disease (CAD) comprises a spectrum of conditions caused by atherosclerotic narrowing of the coronary arteries. Because atherosclerosis progresses slowly from early adulthood, clinical CAD typically manifests in middle and older age [25], consistent with the age range of our participants. In the present study, CAD was confirmed angiographically using the criterion described in the Methods (>50% stenosis in at least one of the three major coronary arteries) [26].

Platelets contribute to CAD at several stages of atherosclerosis, a chronic inflammatory disease in which circulating blood components (platelets and monocytes) and plasma components (lipoproteins) interact with vascular cells [27]. Plaque rupture itself results mainly from inflammation, thinning and weakening of the fibrous cap, degradation of the extracellular matrix and mechanical stress; platelets are principally mediators of the thrombus that forms after plaque disruption, although they also contribute to plaque progression and destabilisation through the inflammatory mediators they release [27], [28]. Moreover, platelet activation in CAD should not be attributed solely to the plaque: systemic inflammation, endothelial dysfunction, disturbed or high shear stress and circulating inflammatory mediators can all promote platelet granule release and increased surface expression of P-selectin and CD40L [19], [28].

Platelets are essential components of the cardiovascular system that contribute significantly to the development and progression of atherosclerosis through their role in thrombosis. Platelet indices, such as Mean Platelet Volume (MPV), Platelet Distribution Width (PDW), and Platelet Large Cell Ratio (P-LCR), are often utilized as markers for platelet activity. Increased platelet activity is associated with an increase in platelet size and volume [29].

MPV is routinely reported by haematology analysers. Larger platelets are generally younger, metabolically and enzymatically more active and more reactive: they contain more dense and α-granules, express more prothrombotic surface receptors such as glycoprotein IIb/IIIa, and synthesise more thromboxane A2 [9], [19]. In CAD, MPV may increase because systemic inflammation, endothelial dysfunction, altered shear stress and thromboinflammatory signalling accelerate platelet activation and consumption; the resulting increase in platelet turnover stimulates megakaryocytes, through thrombopoietin and inflammatory cytokines such as interleukin-6, to release younger and larger platelets [6], [9], [19]. Once activated, platelets undergo shape change, conformational activation of glycoproteins such as GPIIb/IIIa, surface expression of P-selectin and release of more than 300 proteins, including growth factors, chemokines and adhesion ligands [30]. Activated platelets also shed microparticles that expose GPIIb/IIIa and phosphatidylserine, giving them pro-aggregatory and procoagulant capacity; these microparticles can up-regulate endothelial expression of E-selectin, VCAM-1, ICAM-1 and PECAM-1 [15], [29].

MPV and P-LCR provide related but distinct information. MPV is the arithmetic mean volume of the whole platelet population, whereas P-LCR is the proportion of platelets above an analyser-specific volume threshold (12 fL on the analyser used here). P-LCR therefore captures the upper tail of the platelet-volume distribution and may change when a subpopulation of very large platelets expands, even if the mean volume changes little [19], [21]. Both indices are sensitive to preanalytical and analytical conditions: platelets swell progressively in EDTA, so MPV increases with time to analysis, and values differ between anticoagulants and between analysers using impedance or optical methods [19], [31]. In the present study, all samples were collected in K3-EDTA and analysed on the same analyser within 2 h, which reduces, but does not eliminate, this source of variability.

An increase in MPV is related to poor clinical outcomes in CAD patients, as demonstrated by several studies showing a correlation between high MPV values and acute coronary syndrome. Galimzhanov et al. (2022) found that higher admission MPV is associated with increased long-term mortality in ACS patients [32]. Manchanda et al. (2015) showed that increased platelet volume plays a role in the pretrombotic state in CAD, and large platelets contribute to infarction [33]. Suwailem et al. (2020) studies in ST-elevation myocardial infarction have demonstrated that patients with higher mean platelet volume tend to have larger thrombus burden on angiography, indicating more pronounced platelet activation in STEMI [34].

In contrast to several reports linking MPV to the severity and outcome of CAD [32], [33], [34], MPV and P-LCR were not correlated with sVCAM-1 in our cohort, whereas platelet count and absolute large-platelet count were. Several explanations should be considered. First, MPV is primarily a marker of platelet size and turnover and is not necessarily a direct surrogate of endothelial VCAM-1 activity; sVCAM-1 reflects cytokine-driven endothelial activation, which may relate more closely to the total platelet mass available to release CD40L, interleukin-1β and microparticles than to the average size of individual platelets. Second, the absolute large-platelet count is the product of P-LCR and platelet count, yet its association with sVCAM-1 was stronger than that of platelet count and persisted after controlling for platelet count, clinical covariates and leukocyte count, whereas the platelet-count association did not. This suggests that the absolute size of the pool of large, reactive platelets—rather than total platelet number or mean platelet size—tracks endothelial activation, whereas the crude platelet-count association may partly reflect shared systemic inflammation, as platelet and leukocyte counts were correlated. Because MPV and platelet count were inversely correlated in our cohort (ρ = −0.46), as is typical, relative indices such as MPV and P-LCR may not capture an expansion of the large-platelet pool that accompanies a higher platelet count. Third, the MPV range in our cohort was relatively narrow (8.7–13.0 fL), limiting the power to detect a weak correlation. Fourth, all patients had stable CAD, in whom platelet activation and turnover are less pronounced than in acute coronary syndromes; together with antiplatelet and statin therapy and blood sampling shortly after angiography, this may have attenuated any relationship between platelet size and sVCAM-1 [19].

Vascular cell adhesion molecule-1 (VCAM-1) is expressed on the surface of cytokine-activated endothelial cells, and its extracellular domain is shed into the circulation as soluble VCAM-1 (sVCAM-1). In this study we measured the soluble form, which is an indirect, systemic index of endothelial activation rather than a direct measure of VCAM-1 expression within the plaque [35]. VCAM-1 expression is induced by reactive oxygen species, interleukin-1β and tumour necrosis factor-α [15]; hence, increased circulating sVCAM-1 may reflect endothelial activation associated with inflammation, oxidative stress, disturbed flow, diabetes or plaque development [2], [35]. The endothelium is also sensitive to oxidative stress and shear stress, which lead to vascular remodelling and the release of endothelial microparticles; these are shed constantly in healthy individuals and are increased in diseases characterised by endothelial dysfunction [15].

We selected VCAM-1 rather than other endothelial markers for several reasons. ICAM-1 is expressed constitutively at low levels and is also produced by leukocytes and other cell types; E-selectin mainly reflects the initial rolling step of leukocyte recruitment; von Willebrand factor is released from both endothelial cells and platelet α-granules; and endothelin-1 primarily reflects vasomotor tone [2], [35]. In contrast, VCAM-1 is strongly cytokine-inducible, mediates firm adhesion and transendothelial migration of monocytes and lymphocytes through VLA-4, and is expressed early in atherosclerotic lesion formation; in experimental models, VCAM-1—but not ICAM-1—was required for early atherosclerosis [12], [20]. Circulating sVCAM-1 has also been associated with adverse outcomes in patients with CAD [14], [22]. Nevertheless, a panel including ICAM-1, E-selectin and von Willebrand factor would give a more comprehensive picture of endothelial activation and should be considered in future studies.

Leukocyte migration and activation during inflammation depend on interactions between cells and the extracellular matrix [36]. Inflammatory cytokines stimulate endothelial expression of adhesion molecules, proteases and other mediators, some of which enter the bloodstream [35]. Leukocyte recruitment follows a multistep adhesion cascade: P-selectin glycoprotein ligand-1 (PSGL-1) on leukocytes binds endothelial P-selectin and E-selectin, initiating tethering and rolling; leukocyte β2 integrins, particularly LFA-1 (αLβ2) and Mac-1 (αMβ2), then mediate firm adhesion through endothelial ICAM-1; and VCAM-1 binds leukocyte VLA-4 (integrin α4β1), mainly on monocytes and lymphocytes, contributing to firm adhesion and transendothelial migration [16], [20]. P-selectin is the receptor, stored in platelet α-granules and endothelial Weibel–Palade bodies and translocated to the cell surface upon activation, whereas PSGL-1 is its principal ligand on leukocytes [37].

Platelet–leukocyte crosstalk links these pathways. Activated platelets bind leukocytes through P-selectin–PSGL-1, forming platelet–leukocyte aggregates; this interaction promotes leukocyte integrin activation, cytokine and chemokine release and, in neutrophils, the formation of extracellular traps [16], [28]. Activated platelets also release soluble CD40L, which binds CD40 on endothelial cells and induces the expression of ICAM-1, VCAM-1 and E-selectin [17], and deposit chemokines such as RANTES and platelet factor 4 on the endothelium, facilitating monocyte arrest [18]. Through these mechanisms, platelets may contribute to endothelial activation during the initiation of atherosclerosis, to plaque progression through sustained vascular inflammation, and to atherothrombosis after plaque disruption (Fig. 2). However, our cross-sectional data cannot establish whether platelets contribute causally to the sVCAM-1 levels observed.

**Fig. 2:**
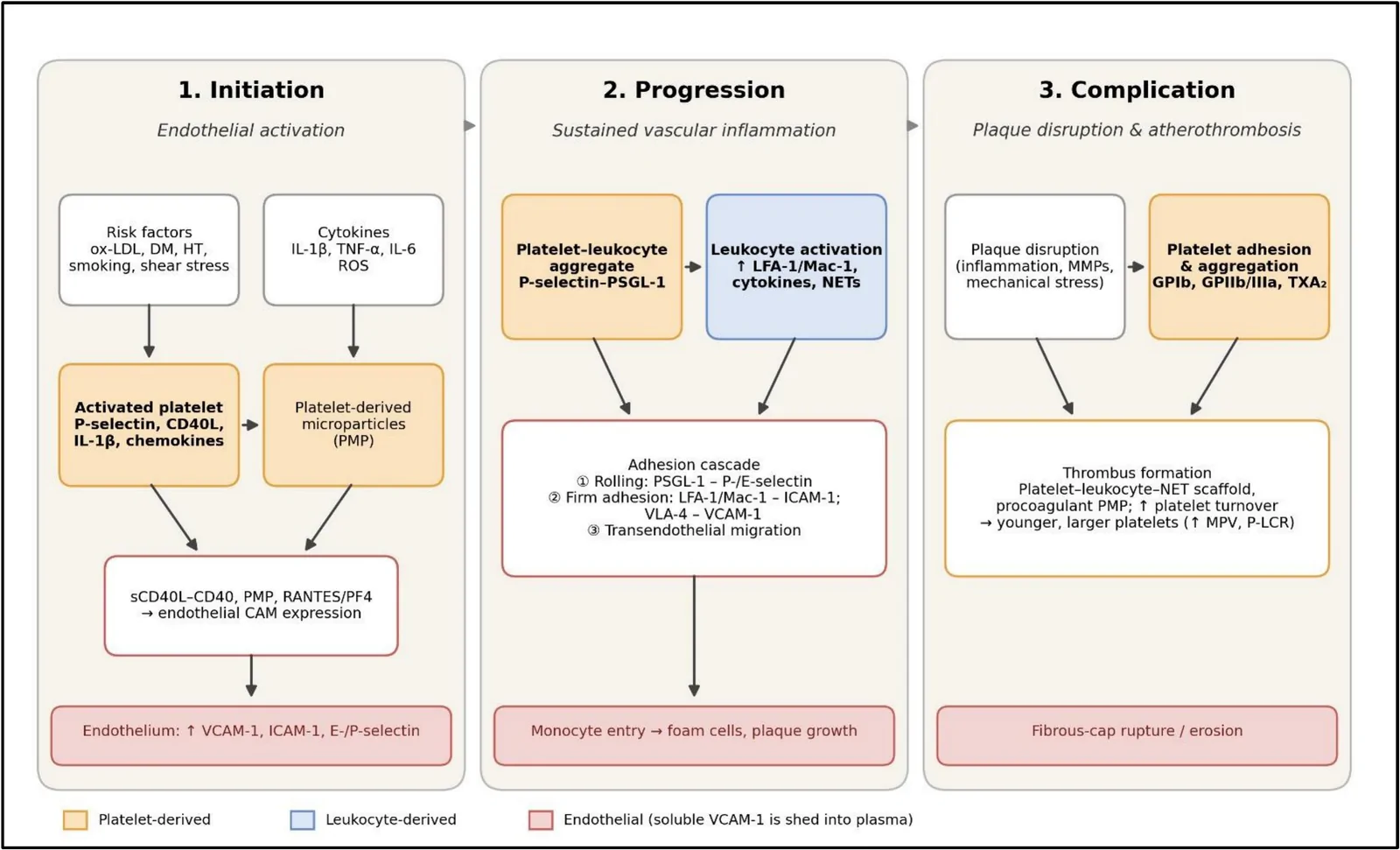
Schematic representation of platelet–leukocyte–endothelial interactions during the initiation, progression and thrombotic complication of atherosclerosis. PSGL-1, P-selectin glycoprotein ligand-1; CD40L, CD40 ligand; VLA-4, very late antigen-4; LFA-1, lymphocyte function-associated antigen-1; Mac-1, macrophage-1 antigen; ICAM-1, intercellular adhesion molecule-1; VCAM-1, vascular cell adhesion molecule-1; PMP, platelet-derived microparticles; NETs, neutrophil extracellular traps.

Normal vascular endothelium is a hallmark of cardiovascular health, and endothelial abnormalities are major contributors to atherosclerosis, ageing, hypertension, obesity and diabetes. Endothelial dysfunction is characterised by an imbalance between vasodilation and vasoconstriction, increased reactive oxygen species, pro-inflammatory signalling and reduced nitric oxide bioavailability [2], [35], and it disrupts endothelial barrier permeability as part of the inflammatory response in cardiovascular disease [38]. Risk factors such as hypertension, oxidised LDL and diabetes increase interleukin-1, interleukin-6, tumour necrosis factor-α and C-reactive protein [39], [40], leading to a pro-inflammatory endothelial phenotype with increased E-selectin, VCAM-1 and ICAM-1 [40], [41]. Increased circulating levels of these molecules have been associated with the severity and outcome of cardiovascular disease [14], [22], although CAMs are produced not only by endothelial cells but also by leukocytes and platelets [42]. In this context, we found that platelet count and absolute large-platelet count were positively correlated with sVCAM-1. These correlations were statistically significant but weak (ρ = 0.24 and ρ = 0.35), corresponding to only about 6% and 12% of shared rank variance, and the platelet-count association did not persist after multivariable adjustment. Platelet count should therefore not be regarded as a strong determinant of VCAM-1, and even the more robust large-platelet association is modest in size. The findings are compatible with coordinated platelet–endothelial and inflammatory activation in CAD, but the cross-sectional design does not allow causal inference, and both variables may simply reflect a shared underlying inflammatory burden. Circulating CAMs signal early inflammation and endothelial activation but have limited diagnostic value when measured alone [35], and neither platelet count nor large-platelet count can currently be used as a clinical biomarker of endothelial dysfunction or CAD; any clinical implication of our findings is hypothesis-generating and requires prospective validation.

This study has several limitations. First, the cross-sectional design precludes causal inference regarding the relationship between platelet indices and sVCAM-1. Second, the sample size was modest (*n* = 80) and derived from a single centre, which limits statistical power, particularly for detecting weak correlations, and generalisability; selection bias cannot be excluded. Third, platelet indices and sVCAM-1 were measured at a single time point, so biological variability and changes over the course of disease could not be assessed. Fourth, diabetes, hypertension, obesity, smoking, dyslipidaemia, chronic kidney disease, infection, chronic inflammatory disease and even vitamin D deficiency can independently influence platelet indices, platelet activation and VCAM-1 [19], [43], [44], as can antiplatelet and statin therapy; although the main associations were adjusted for available covariates, a formal diagnosis of hypertension and dyslipidaemia was not recorded, lipid profiles and HbA1c were incomplete, all patients received statins, and residual confounding is therefore likely. Fifth, only patients with stable CAD were included, so the findings cannot be extrapolated to acute coronary syndromes, in which platelet activation and VCAM-1 may differ substantially; moreover, blood was drawn on the day of or the day after angiography, and procedural heparin, contrast exposure or antiplatelet loading may have influenced platelet indices. Sixth, platelet indices are only indirect markers of platelet activation; we did not measure direct platelet-activation markers (e.g., P-selectin, soluble CD40L), inflammatory biomarkers such as C-reactive protein, platelet–leukocyte aggregates or circulating microparticles. Seventh, sVCAM-1 was measured in serum rather than microparticle-free plasma, so residual endothelial- and platelet-derived microparticles may have influenced the measured concentration [45]; serum was stored at −20 °C rather than −80 °C for up to 5 months, and samples were assayed in singlicate, which precludes estimation of within-run imprecision. Finally, there was no non-CAD control group, and plaque burden was not quantified with an objective score (e.g., SYNTAX or Gensini score). Future prospective studies with larger cohorts, longitudinal follow-up, adjustment for comorbidities and medications, and direct assessment of platelet activation, inflammatory biomarkers, platelet-leukocyte aggregates, microparticles and objective measures of plaque burden are warranted.

## 5 Conclusion

In patients with stable, angiographically confirmed CAD, absolute large-platelet count and platelet count showed statistically significant but weak positive correlations with circulating sVCAM-1, whereas MPV and P-LCR did not; only large-platelet count remained associated after multivariable adjustment. These findings suggest a modest association between the large-platelet pool and endothelial activation but do not establish causality or support the use of these indices as clinical biomarkers. Larger prospective studies with adjustment for comorbidities and direct assessment of platelet, leukocyte, endothelial and microparticle activation are needed.

## CRediT authorship contribution statement

**Ariosta:** Writing – original draft, Resources, Investigation. **Banundari Rachmawati:** Supervision. **Udin Bahrudin:** Supervision. **N.S. Widyastiti:** Supervision. **Dwi Retnoningrum:** Supervision.

## Ethics declaration

### Informed consent and patient details

Written informed consent to take part in the study and to publish the article has been obtained from all participants or their legal representatives. The privacy rights of participants have been observed.

### Human and biological material

This study included organ or tissue donors.

This study includes human biological material and consent was obtained by donors, or their next of kin or legal representatives, for use in this study and for publication of the article. The samples used in this research were not sourced from executed prisoners or prisoners of conscience.

### Studies in human

This study was performed in compliance with relevant laws, regulatory frameworks and guidelines where the research took place.

This study was approved by the Health Research Ethics Committee RSUP Dr. Kariadi Semarang.

(Approval No. 791/EC/KEPK-RSDK/2021)

## Declaration of competing interest

The authors declare the following financial interests/personal relationships which may be considered as potential competing interests:

Ariosta reports financial support was provided by Diponegoro University Faculty of Medicine. Reports a relationship with that includes: Has patent pending to. If there are other authors, they declare that they have no known competing financial interests or personal relationships that could have appeared to influence the work reported in this paper.
